# Predictive value of prognostic nutritional and systemic immune-inflammation indices for patients with microsatellite instability-high metastatic colorectal cancer receiving immunotherapy

**DOI:** 10.3389/fnut.2023.1094189

**Published:** 2023-05-18

**Authors:** Jiahong Yi, Ju Xue, Lin Yang, Liangping Xia, Wenzhuo He

**Affiliations:** ^1^Department of VIP Region, Collaborative Innovation Center for Cancer Medicine, State Key Laboratory of Oncology in South China, Sun Yat-sen University Cancer Center, Guangzhou, China; ^2^Department of Radiation Oncology, Nanfang Hospital, Southern Medical University, Guangzhou, Guangdong, China

**Keywords:** prognostic nutritional indeximmunotherapy, mCRC, prognosis, survival, systemic immune-inflammation index

## Abstract

**Background:**

The prognostic nutritional index (PNI) and systemic immune-inflammation index (SII) are indicators of nutritional immune status. They have been reported associated with clinical outcomes of various solid tumors. However, it is unclear whether they can serve as predictors for patients with microsatellite instability-high (MSI-H) metastatic colorectal cancer (mCRC) receiving immunotherapy. Our objective was to study the prognostic value of PNI and SII in these patients.

**Methods:**

Seventy-five MSI-H mCRC patients were enrolled in our study. Logistic regression analysis was used to identify features that influenced immunotherapy response. Survival differences between groups of mCRC patients were compared using the Kaplan–Meier method and log-rank test. The independent risk parameters for progression-free survival (PFS) and overall survival (OS) of patients with MSI-H mCRC were established by Cox proportional risk regression analysis.

**Results:**

The optimal SII and PNI cutoff values were 409.6 and 51.35. Higher PNI (*p =* 0.012) and lower high-density lipoprotein cholesterol (HDLC, *p* = 0.012) were associated with a better immunotherapy response. SII (*p* = 0.031), cholesterol (CHO) (*p* = 0.007) and aspartate aminotransferase (AST) (*p* = 0.031) were independent prognostic factors correlated with OS. Higher PNI (*p* = 0.012) and lower AST (*p* = 0.049) were negative predictors of PFS. In addition, patients suffered from immune-related adverse events (irAEs) had a lower SII level (*p* = 0.04).

**Conclusion:**

Higher AST and SII, and lower PNI predict worse outcomes in MSI-H mCRC patients undergoing immunotherapy. Moreover, patients with lower SII before immunotherapy suffered from irAEs more often.

## 1. Introduction

A significant portion of cancer-related mortality is caused by colorectal cancer (CRC), which is not only the most common cancer-related death worldwide but also one of the most prevalent cancer-related death types (1). Approximately 25% patients with CRC are diagnosed with advanced-stage disease, and 25–50% patients with early-stage cancer will develop metastasis (2). Despite the continuous optimization of CRC prevention and treatments, the incidence and mortality of CRC are still increasing (3). Approximately 15% of the patients were microsatellite instability-high (MSI-H) among all CRC patients, and 5% patients with MSI-H CRC are metastatic CRC (mCRC) (4). Programmed death-1 (PD-1) checkpoint blockades have significantly improved the survival of MSI-H mCRC (5, 6). The KEYNOTE-177 study concluded Pembrolizumab as first-line therapy improved progression-free survival (PFS) in patients with dMMR/MSI-H mCRC compared to chemotherapy combined with bevacizumab/cetuximab (7). Pembrolizumab improved ORR from 43.8 to 45% in the final analysis. Immunotherapy has dramatically changed the management of CRC (8). The ORR ranged from 28 to 52% when patients with dMMR/MSI-H mCRC treated with PD-1 blockade (7, 9, 10). Approximately half patients could not benefit from immunotherapy. The better predictive biomarkers were urgently required to identify immunotherapy-eligible patients.

Some studies have shown the relationship between systemic immune-inflammatory makers and earlier identification of different tumors and the association between systemic inflammation markers and prognosis (11). Over the past few years, the prognostic nutritional index (PNI) has been investigated as a prognostic marker in various tumors (12–14). For example, gastrointestinal tumors with lower PNI had a worse prognosis (15). The systemic immune-inflammation index (SII) was shown to be a useful prognostic indicator in patients with pancreatic cancer (16), gastroesophageal adenocarcinoma (17), invasive vulvar cancer (18) and lung cancer (19, 20). It was reported that low PNI was associated with a worse immunotherapy response in patients with advanced cancer. There were many kinds of cancers in this research, such as lung cancer, melanoma and so on (21). As an immune-inflammation biomarker, SII did not appear to be a significant predictor in patients suffered from advanced melanoma treated with immunotherapy (22). However, the predictive role of PNI and SII in MSI-H mCRC patients treated with immunotherapy is still unclear. Therefore, we retrospectively collected these patients’ data to analyze the predictive prognostic effect of baseline inflammatory indicators on immunotherapy efficacy.

## 2. Materials and methods

### 2.1. Patients

Seventy-five newly diagnosed MSI-H mCRC patients treated with anti-PD1 at Sun Yat-sen University Cancer Center between June 2017 and June 2021 were retrospectively analyzed. The inclusion criteria were: (1) pathologically diagnosed with CRC confirmed as MSI-H by Next Generation Sequencing (NGS), (2) diagnosis of stage IV unresectable disease according to the 8th edition of the American Joint Committee on Cancer (AJCC), (3) age ≥ 18 years at diagnosis, (4) at least 3 months of follow-up, (5) received anti-PD-1 therapy with or without other therapy, anti-PD-1 therapy contained Nivolumab, Pembrolizumab, Camrelizumab, Sintilimab or Toripalimab. The exclusion criteria were: (1) chronic inflammatory or rheumatological disease, (2) having a known diagnosis of Diabetes Mellitus (DM) and using drugs that can affect the Fasting Blood Glucose (FBG), and (3) incomplete clinicopathological data.

### 2.2. Data collection

Electronic medical records were reviewed to retrieve clinical and laboratory data at baseline. Clinicopathological characteristics data included gender, age, immunotherapy regimen, liver metastasis status, and lung metastasis status. Within 1 week of immunotherapy, blood laboratory investigations and biochemical indices were collected: blood cell count (cell/μL), hemoglobin (gr/dL), platelet count (cell/μL), bilirubin (mg/dL), high-density lipoprotein cholesterol (HDLC; mmol/L), C-reactive protein (CRP; mg/L), cholesterol (CHO; mmol/L), aspartate aminotransferase (AST; IU/L), alanine aminotransferase (ALT; IU/L), and albumin (g/dL). The values of PNI, SII, and Lymphocyte-C-reactive Protein Ratio (LCR) were calculated according to the following formula: PNI = serum albumin (g/L) + 5 × peripheral blood lymphocyte count (×10^9^ /L), LCR = Lymphocyte/C-reactive Protein, SII = (platelet count) × (the neutrophil-to-lymphocyte ratio).

Medical Ethics Committee approval was granted (GZR2023-146) to the study by Sun Yat-sen University Cancer Center.

### 2.3. Follow-up

Every patient was followed-up with regularly until August 2022 or death. A follow-up examination was conducted after immunotherapy every 3 months for the first year, every 6 months for the next 2 years, and every year after that. The curative effect was evaluated according to RECIST evaluation criteria. There were four response categories: complete remission (CR), partial remission (PR), disease stability (SD), and disease progression (PD). PFS was measured from the date of the initial pathological diagnosis until the date of PD, death or last follow-up (months). Overall survival time (OS) was determined by calculating the months between the date of diagnosis and death or last follow-up.

### 2.4. Statistical analysis

**Figure 1:**
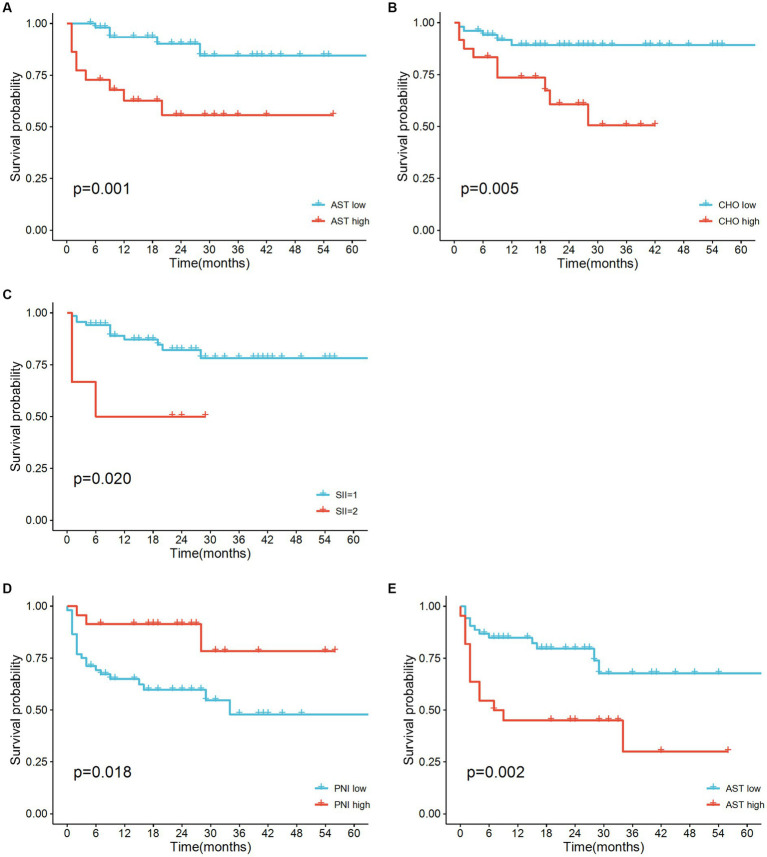
Comparison of SII ratio levels according to the onset of irAEs.

The numbers (%) were presented as categorical data, while the means and standard deviations (SD) were presented as continuous data. Categorical variables were analyzed using chi-square tests or Fisher’s exact tests, while continuous variables were analyzed using *t*-tests. The optimal cutoff values were confirmed by ROC curves predicted OS. The correlation between peripheral blood markers and immunotherapy response was investigated using logistic regression. PFS and OS curves were calculated by using the Kaplan–Meier method, and differences were assessed by log-rank test. PFS and OS independent indicators were identified by cox regression models. Multivariate analysis was conducted using statistically significant factors in the univariate analysis. Interaction analyzes were used to investigate the association between PNI or SII with various clinical parameters. *p*-values <0.10 were used for interaction analyzes and value of *p* <0.05 was considered statistically significant in other analyzes. All statistical tests were conducted using SPSS 27.0 (SPSS, Chicago, IL). Figure 1 were made using GraphPad Prism 9.0.

## 3. Results

### 3.1. Clinicopathological characteristics of patients with MSI-H mCRC received immunotherapy

Our study enrolled 75 patients with MSI-H mCRC who were undergoing immunotherapy. ROC analysis was conducted, using cancer-specific death as an endpoint, to determine the optimal cutoff point with the Youden index. The maximal cutoffs were 51.35, 409.6, 1.03, 4.88, 1.15, 9.9, 28.2, 1810.78 for PNI, SII, CRP, CHO, HDLC, ALT, AST, LCR, respectively.

The median age was 47 years (range 23–84), and 27 (36%) patients were female. Table 1 demonstrated the demographic, clinical, and pathological characteristics of patients. The median follow-up time was 24 (95% CI: 19.31 to 28.69) months. The ORR was 41.33% [31/75, 10 complete responses (CR), 21 partial responses (PR)]. The disease control rate (DCR) was 86.7% [58/75, 10 CR, 21 PR, 34 stable diseases (SD)].

**Table 1:** Patients characteristics before immunotherapy.

Title	Category	Number	Percentage (%)
Sex	Female	27	36
	Male	48	64
Age	< 60 years	59	78.7
	≥ 60 years	16	21.3
Site	Left	39	52
	Right	34	45.3
	Both	2	2.7
Lynch syndrome	No	41	54.7
	Yes	34	45.3
Liver metastasis	No	51	68
	Yes	24	32
Lung metastasis	No	66	88
	Yes	9	12
Immunotherapy type	Anti-PD1	70	93.3
	Anti-PD1 + anti-CALT4	5	6.7
Chemotherapy	No	54	72
	Yes	21	28
Anti-angiogenesis	No	63	84
	Yes	12	16
Line	1	51	68
	≥ 2	24	32
Best efficacy	CR	10	13.3
	PR	21	28
	SD	34	45.3
	PD	10	13.3
CRP	Low	13	17.3
	High	62	82.7
CHO	Low	51	68
	High	24	32
HDLC	Low	38	50.7
	High	37	49.3
ALT	Low	17	22.7
	High	58	77.3
AST	Low	53	70.7
	High	22	29.3
PNI	Low	52	69.3
	High	23	30.7
LCR	Low	42	56
	High	33	44
SII	Low	69	92
	High	6	8

CR, complete remission; PR partial remission; SD, disease stability; PD, disease progression; CRP, C-reactive protein; CHO, cholesterol; HDLC, high density lipoprotein cholesterol; ALT, alanine aminotransferase; AST, aspartate aminotransferase; PNI, prognostic nutritional index; LCR, Lymphocyte-C-reactive Protein Ratio; SII, systemic immune-inflammation index.

### 3.2. Factors of influenced immunotherapy response in patients with MSI-H mCRC

The baseline clinicopathological characteristics were used to assess patients’ response to immunotherapy. There were significant differences in HDLC, AST, and PNI (*p* < 0.05) (as shown in Supplementary Table S1). Univariate analysis showed that HDLC (*p =* 0.014) and PNI (*p =* 0.028) were significantly associated with clinical response. The multivariate regression model showed that lower levels of HDLC and higher PNI were independent risk factors for the clinical benefit (OR = 4.709, 95% CI 1.415–15.666, *p* = 0.012; OR = 0.162, 95% CI 0.032–0.951, *p* = 0.815, respectively; Table 2).

**Table 2:** Univariate and multivariate analyzes of biomarkers for immunotherapy response.

Variables	Category	Univariate analysis				Multivariate analysis			
		OR	OR (95% CI)		*p* value	OR	OR (95% CI)		*p* value
Sex	Male	1.416	0.488	4.111	0.522				
Age	>60 years	0.471	0.144	1.54	0.213				
Site	Right	2.523	0.877	7.257	0.086				
Lynch syndrome	Yes	1.596	0.548	4.65	0.392				
Liver metastasis	Yes	0.55	0.187	1.619	0.278				
Lung metastasis	Yes	1.214	0.23	6.421	0.819				
Immunotherapy type	Anti-PD1 + anti-CALT4	0.481	0.074	3.124	0.443				
Chemotherapy	Yes	2.526	0.652	9.791	0.18				
Anti-angiogenesis	Yes	1.848	0.367	9.308	0.457				
Line	≥2	0.55	0.187	1.619	0.278				
CRP	High	0.481	0.097	2.4	0.372				
CHO	High	0.407	0.138	1.194	0.102				
HDLC	High	4.017	1.270	12.708	0.018	4.709	1.415	15.666	0.012
ALT	High	0.322	0.066	1.561	0.159				
AST	High	0.336	0.113	1.003	0.051				
PNI	High	5.1	1.07	24.315	0.041	0.162	0.032	0.815	0.027
LCR	High	1.109	0.387	3.176	0.847				
SII	High	0.654	0.11	3.891	0.641				

### 3.3. Univariate and multivariate analyzes of biomarkers for OS and PFS

**Figure 2:**
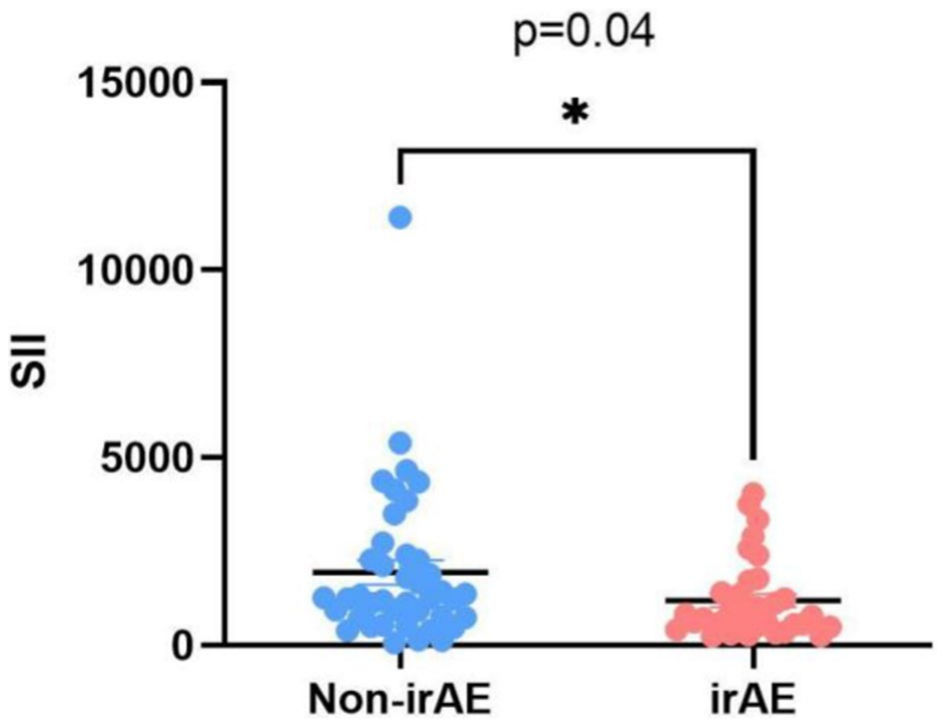
Kaplan–Meier survival curves for overall survival **(A–C)** and disease-free survival **(D–E)** for patients with MSI-H mCRC with high versus low SII, PNI, AST and CHO, respectively.

According to univariate analysis, patients with liver metastasis (*p* = 0.031), higher AST (*p* = 0.004), higher SII (*p* = 0.032), and higher CHO (*p* = 0.01) predicted shorter OS. Female patients (*p* = 0.049), as well as patients with higher PNI (*p* < 0.03), had longer PFS after immunotherapy, while higher AST (*p* = 0.005) were identified as negative factors for predicting PFS. The Cox regression model verified AST, SII, and CHO as independent prognostic factors for OS (Table 3). PNI was verified as an independent prognostic factor for PFS and AST (Table 4). Median OS was shorter in elevated SII (Not Reached vs. 6.0 months, *p* = 0.031) and reduced AST group (Not Reached vs. Not Reached, *p* = 0.031). PFS was shorter in shown in higher PNI (Not Reached vs. 34 months, *p* = 0.049) and lower AST (Not Reached vs. 7.0 months, *p* = 0.012), as shown in Figure 2. Interaction analyzes revealed that no variable had any obvious influence on the association between SII and OS in our study (Supplementary Table S2). Higher PNI was significantly associated with shorter PFS for the following factors: sex, Immunotherapy type and LCR (all *P* for interaction <0.1, Supplementary Table S3).

**Table 3:** Univariate and multivariate analyzes of biomarkers for OS.

Variables	Category	Univariate analysis				Multivariate analysis			
		HR	95% CI	*p* value		HR	95% CI		*p* value
Sex	Female	2.058	0.72	5.882	0.178				
Age	<60 years	1.618	0.507	5.165	0.417				
site	Left	0.388	0.126	1.193	0.098				
Lynch syndrome	No	0.931	0.322	2.691	0.895				
Liver metastasis	No	0.312	0.108	0.902	0.031	0.931	0.238	3.645	0.918
Lung metastasis	No	0.488	0.136	1.752	0.272				
Immunotherapy type	No	0.471	0.105	2.109	0.325				
Chemotherapy	Anti-PD1	1.206	0.377	3.861	0.752				
Anti-angiogenesis	No	0.757	0.211	2.721	0.67				
Line	1	0.458	0.16	1.312	0.146				
CRP	Low	0.033	0	7.475	0.217				
CHO	Low	0.239	0.08	0.713	0.01	0.173	0.049	0.613	0.007
HDLC	Low	1.792	0.6	5.353	0.296				
ALT	Low	1.814	0.406	8.117	0.436				
AST	Low	0.200	0.067	0.596	0.004	0.182	0.039	0.854	0.031
PNI	Low	0.158	0.021	1.207	0.075				
LCR	Low	0.298	0.083	1.071	0.064				
SII	Low	0.247	0.069	0.89	0.032	0.182	0.039	0.854	0.031

**Table 4:** Univariate and multivariate analyzes of biomarkers for PFS.

Variables	Category	Univariate analysis				Multivariate analysis			
		HR	HR (95% CI)		*p* value	HR	HR (95% CI)		*p* value
Sex	Female	0.453	0.206	0.995	0.049	0.662	0.29	1.509	0.12
Age	<60 years	1.477	0.616	3.54	0.382				
Site	Left	0.725	0.35	1.501	0.386				
Lynch syndrome	No	0.836	0.374	1.872	0.664				
Liver metastasis	No	0.468	0.212	1.031	0.06				
Lung metastasis	No	1.058	0.316	3.542	0.927				
Chemotherapy	No	0.513	0.192	1.375	0.185				
Immunotherapy type	Anti-PD1	2.124	0.635	7.109	0.222				
Anti-angiogenesis	No	1.243	0.466	3.316	0.664				
Line	1	1.968	0.896	4.323	0.092				
CRP	Low	3.293	0.763	14.217	0.11				
CHO	Low	0.463	0.211	1.016	0.055				
HDLC	Low	1.556	0.695	3.481	0.282				
ALT	Low	2.398	0.717	8.02	0.156				
AST	Low	3.114	1.417	6.844	0.005	0.291	0.085	0.993	0.049
PNI	Low	0.263	0.079	0.88	0.030	0.353	0.156	0.797	0.012
LCR	Low	0.528	0.227	1.228	0.138				
SII	Low	0.478	0.142	1.608	0.233				

### 3.4. Pretreatment peripheral blood parameters and the incidence of irAEs

The number of patients with irAEs was 45.3%. They were all grade 1 or 2 irAEs. The most observed irAEs were diarrhea (12.0%), hepatic toxicity (9.3%), and oral mucositis or rash (5.3%). The detailed toxicity spectrum is provided in Supplementary Table S2. The incidence of irAEs was correlated with SII levels. As shown in Figure 1, SII was lower in patients with irAEs (*p* = 0.04).

## 4. Discussion

Although microsatellite instability status is a biomarker for selecting mCRC patients suitable for immunotherapy. Only almost 50% of MSI-H mCRC patients benefited from immunotherapy and some even experienced hyper-progression, leading to worse survival (23). It is necessary to find effective predictors for patients with mCRC treated by immunotherapy. TMB also was used to screen appropriate patients received immunotherapy. But patients with mCRC generally have lower TMB than other cancers (24). These factors limited physicians to select potentially MSI-H mCRC patients beneficial from immunotherapy. Biomarkers are needed to identify the subset of mCRC patients who are benefit from immunotherapy (25).

This is the first study to explore prognostic factors of immunotherapy in MSI-H mCRC patients to date. Due to the small proportion of overall colorectal cancer patients. The prognostic value of peripheral blood indicators in these patients is unknown, and little is known about their prognostic value. Our results indicate a significant association between higher SII and poorer OS. This finding suggests that baseline SII plays a significant role in the progression of MSI-H mCRC. We also found that a higher baseline PNI status was associated with longer PFS and patients with elevated baseline PNI were more suitable for immunotherapy. By evaluating baseline PNI, it is possible to select appropriate patients to receive immunotherapy. Furthermore, Patients developed irAEs more easily when their SII increased. Higher SII may be related to the occurrence of irAEs, which needs to be further explored.

In recent years, there has been increasing evidence that peripheral blood inflammatory indicators, nutritional indicators, and some indicators derived from them are related to the prognosis of patients suffering from advanced cancer. Patients with various cancers have prognostic significance based on their nutritional status and inflammation (12, 16, 26). SII was a powerful prognostic indicator for patients with pancreatic, gastric, and lung cancer (27–30). Apart from this, these inflammatory indicators can predict the effectiveness of antitumor therapy in oncology patients. For example, it was reported that SII was an independent prognostic factor in patients with mCRC who received chemotherapy with or without bevacizumab, and the lymphocytic response to the tumor was associated with it (31, 32). SII contains three types of inflammatory cells, neutrophil, platelet and lymphocyte. Among them, neutrophil and platelet can promote cancer cells proliferation and metastasis via multiple mechanisms (33, 34). Lymphocyte may produce a favorable microenvironment for tumor infiltration (35). SII was significantly associated with TIL in tumor microenvironment. Cytotoxic T lymphocytes (CTLs) inhibit tumor growth by secreting anti-angiogenic factors and cytokines that induce apoptosis of tumor cells (36). Tumor-infiltrating lymphocytes are associated with better OS in patients with tumors. The specific mechanism between the inflammatory state of the body associated with SII and changes in TIL in the tumor microenvironment needs to be further explored.

Published data demonstrated an obvious correlation between PNI and the prognosis of some tumors, including biliary (37), oral (38), and lung cancer recently (39). In addition, PNI is associated with the response to immunotherapy (40–42). In other words, lower PNI contributed to higher risk of disease progression and poor outcomes. PNI plays an important part in helping clinicians to decide whether to give adjuvant chemotherapy to CRC patients after surgery. In stage III CRC patients, A decreased PNI was an independent factor result in a poor prognosis. But OS and DFS could been ameliorated if patients received 6–8 cycles of adjuvant chemotherapy (43). A lower PNI is a risk factor for obstructive CRC among surgically treated CRC patients (44). Lymphocytes participated in body’s immune regulation and destroyed tumor cells through cellular and humoral immunity (45). Albumin is predominant protein in human plasma and maintains the body’s nutrition and osmotic pressure. Numerous studies have shown that nutritional status is closely related to immune function and that changes in cellular metabolism affect immune cell function (46). PNI is a new method for assessing the immune and nutritional status of patients based on serum lymphocyte counts and albumin levels. Thus, PNI may be a valid predictor for immunotherapy in cancer patients. However, no studies have evaluated its predictive role in patients with MISH mCRC receiving immunotherapy. Our research confirmed that lower levels of PNI predicted worse outcomes. On the other hand, peripheral blood index testing is convenient and inexpensive. It is widely used in clinic and has great clinical significance for clinicians to select patients suitable for immunotherapy.

Interestingly, we discovered the role of AST in predicting the outcome of immunotherapy in our patients. Univariate analysis found that liver metastases were associated with a poor prognosis of OS, which had also been confirmed in other tumors (47, 48). Higher AST levels possibly was an independent factor to predict poor PFS, OS, and response to immunotherapy. This suggested the potential role of the liver as an immune organ in influencing the effectiveness of immunotherapy in liver metastasis. It had been confirmed that macrophages and Treg cells increased in liver metastasis of mCRC controlled systemic immunity and immunotherapy response (49, 50). AST was released into the blood when hepatocytes were damaged (51). In healthy individuals, the level of AST in blood is very low. Elevated AST reflected the progression of HCC (52). AST predicted chronic hepatitis B virus immune tolerance when combined with HBcAb in Zhang’s study (53). As a common indicator of liver function, AST probably could be used as a simple indicator to predict the effect of immunotherapy. However, the role of AST in influencing the efficacy of immunotherapy requires more attention. Lower HDLC was independent risk factors for the clinical benefit in our study. Wang et al. found preoperative lower HDL-C present with poor prognosis in stage II/III CRC patients regardless of MSI status (54). As is known to all, patients with different MSI status respond differently to immunotherapy (55), and the immune status varied with different MSI status. Potential interaction mechanisms between HDLC and immune function worth further exploration in different kinds of CRC patients.

Concerning the limitations of our study, it is retrospective research and the number of enrolled patients is small, which may induce selection bias and limit the generalizability of the results. Secondly, enrolled patients’ treatment protocols were not uniform. Some patients received anti-PD-1 in the second line treatment or beyond; others received immunotherapy with chemotherapy or anti-angiogenesis drugs. It might be confounding factors that influenced the conclusion. In addition, there is a need for further validation of the predictive value of the peripheral blood markers (AST, SII, and PNI) in terms of OS, PFS, or irAEs by randomized controlled trial. Despite these limitations, our study was unique because PNI and SII combined three baseline markers of peripheral blood. In addition, to our knowledge, this is the first study to explore the correlation between peripheral blood markers (SII, PNI, and AST) in patients with MSHI mCRC who accepted anti-PD-1 treatment. These findings enhance the understanding that multiple information, including baseline peripheral blood parameters, clinical outcomes, and irAEs in MSI-H mCRC patients receiving immunotherapy-based treatment. The findings tell us MSI-H mCRC patients’ nutritional and inflammatory status may be prognostic factors for immunotherapy. Secondly, there may be a relationship between irAEs and excessive inflammatory response. We can use these indicators extensively to select patients who will benefit from immunotherapy due to their low cost and ease of detection.

In conclusion, our study suggested higher AST, higher SII, and lower levels of PNI predicted worse outcomes in MSI-H mCRC patients undergoing immunotherapy. Patients with lower SII before immunotherapy suffered from irAEs more easily. This provided reference for physicians to identify patients who can benefit from immunotherapy.

## Data availability statement

The original contributions presented in the study are included in the article/Supplementary material, further inquiries can be directed to the corresponding authors.

## Ethics statement

The studies involving human participants were reviewed and approved by Medical Ethics Committee approval was granted (B2020-256) to the study by Sun Yat-sen University Cancer Center. Written informed consent for participation was not required for this study in accordance with the national legislation and the institutional requirements.

## Author contributions

JY and JX conducted and drafted the manuscript. JY collected and analyzed the data. LY and WH designed the manuscript. LX and WH revised the manuscript. All authors listed have made a substantial, direct, and intellectual contribution to the work and approved it for publication.

## Funding

This study was supported by the National Science Fund for Distinguished Young Scholars of China (82002557).

## Conflict of interest

The authors declare that the research was conducted in the absence of any commercial or financial relationships that could be construed as a potential conflict of interest.

## Publisher’s note

All claims expressed in this article are solely those of the authors and do not necessarily represent those of their affiliated organizations, or those of the publisher, the editors and the reviewers. Any product that may be evaluated in this article, or claim that may be made by its manufacturer, is not guaranteed or endorsed by the publisher.
