# Supplementary material for: Predictive value of prognostic nutritional and systemic immune-inflammation indices for patients with microsatellite instability-high metastatic colorectal cancer receiving immunotherapy
Source: Front Nutr. 2023 May 18;10:1094189. doi: 10.3389/fnut.2023.1094189 (PMC10232767; doi:10.3389/fnut.2023.1094189)
Supplement: Supplementary file 1 [file Data_Sheet_1.docx]

supplementary materials

**Table 1 Factors associated with [immunotherapy response](https://pubmed.ncbi.nlm.nih.gov/34101624/) in patients with MSIH mCRC**

| Variables | Category | Response | | Total | P value |
| --- | --- | --- | --- | --- | --- |
|  |  | no | yes |  |  |
| sex | female | 8 | 19 | 27 | 0.521 |
|  | male | 11 | 37 | 48 |  |
| age | <60 years | 13 | 46 | 59 | 0.207 |
|  | >60 years | 6 | 10 | 16 |  |
| site | left | 13 | 26 | 39 | 0.217 |
|  | right | 6 | 28 | 34 |  |
| lynch syndrome | no | 12 | 29 | 41 | 0.39 |
|  | yes | 7 | 27 | 34 |  |
| Liver metastasis | no | 11 | 40 | 51 | 0.274 |
|  | yes | 8 | 16 | 24 |  |
| Lung metastasis | no | 17 | 49 | 66 | 0.819 |
|  | yes | 2 | 7 | 9 |  |
| Immunotherapy type | anti-PD1 | 17 | 53 | 70 | 0.435 |
|  | anti-PD1 +anti-CALT4 | 2 | 3 | 5 |  |
| chemotherapy | no | 16 | 38 | 54 | 0.17 |
|  | yes | 3 | 18 | 21 |  |
| Anti-angiogenesis | no | 17 | 46 | 63 | 0.451 |
|  | yes | 2 | 10 | 12 |  |
| line | 1 | 11 | 40 | 51 | 0.274 |
|  | ≥2 | 8 | 16 | 24 |  |
| CRP | low | 2 | 11 | 13 | 0.364 |
|  | high | 17 | 45 | 62 |  |
| CHO | low | 10 | 41 | 51 | 0.097 |
|  | high | 9 | 15 | 24 |  |
| HDLC | low | 5 | 33 | 38 | **0.014** |
|  | high | 14 | 23 | 37 |  |
| ALT | low | 2 | 15 | 17 | 0.144 |
|  | high | 17 | 41 | 58 |  |
| AST | low | 10 | 43 | 53 | **0.046** |
|  | high | 9 | 13 | 22 |  |
| PNI | low | 17 | 35 | 52 | **0.028** |
|  | high | 2 | 21 | 23 |  |
| LCR | low | 11 | 31 | 42 | 0.847 |
|  | high | 8 | 25 | 33 |  |
| SII | low | 17 | 52 | 69 | 0.639 |
|  | high | 2 | 4 | 6 |  |

**Table 2** No variable had any obvious influence on the association between SII and OS

| Variables | p value for interaction |
| --- | --- |
|  |  |
| sex | 0.359 |
| age | 0.816 |
| site | 0.53 |
| lynch syndrome | 0.223 |
| Liver metastasis | 0.596 |
| Lung metastasis | 0.462 |
| Immunotherapy type | 0.332 |
| chemotherapy | 0.379 |
| Anti-angiogenesis | 0.711 |
| line | 0.962 |
| CRP | 0.902 |
| CHO | 0.525 |
| HDLC | 0.404 |
| ALT | 0.317 |
| AST | 0.348 |
| PNI | 0.688 |
| LCR | 0.26 |

**Table 3** higher PNI was signifificantly associated with shorter PFS for sex, Immunotherapy type and LCR

| Variables | p value for interaction |
| --- | --- |
|  |  |
| sex | **0.069** |
| age | 0.571 |
| site | 0.592 |
| lynch syndrome | 0.41 |
| Liver metastasis | 0.896 |
| Lung metastasis | 0.345 |
| chemotherapy | 0.402 |
| Immunotherapy type | **0.06**3 |
| Anti-angiogenesis | 0.443 |
| line | 0.924 |
| CRP | 0.724 |
| CHO | 0.598 |
| HDLC | 0.826 |
| ALT | 0.507 |
| AST | 0.436 |
| LCR | **0.035** |
| SII | 0.593 |

**Table 4 Distribution of immune-related adverse events**

| Variables | Category | number | Percentage(%) |
| --- | --- | --- | --- |
| irAEs | no | 41 | 54.7 |
|  | yes | 34 | 45.3 |
| oral mucositis or rash | yes | 4 | 5.3 |
| diarrhea | yes | 9 | 12.0 |
| Alanine aminotransferase or bilirubin increase | yes | 7 | 9.3 |
| pneumonia | yes | 3 | 4.0 |
| Immune-associated thyroiditis | yes | 3 | 4.0 |
| immune-related encephalitis | yes | 1 | 1.3 |

irAEs, immune-related adverse events
